# The *Anopheles gambiae* Oxidation Resistance 1 (OXR1) Gene Regulates Expression of Enzymes That Detoxify Reactive Oxygen Species

**DOI:** 10.1371/journal.pone.0011168

**Published:** 2010-06-17

**Authors:** Giovanna Jaramillo-Gutierrez, Alvaro Molina-Cruz, Sanjeev Kumar, Carolina Barillas-Mury

**Affiliations:** Laboratory of Malaria and Vector Research, National Institute of Allergy and Infectious Diseases, National Institutes of Health, Rockville, Maryland, United States of America; New York University School of Medicine, United States of America

## Abstract

**Background:**

OXR1 is an ancient gene, present in all eukaryotes examined so far that confers protection from oxidative stress by an unknown mechanism. The most highly conserved region of the gene is the carboxyl-terminal TLDc domain, which has been shown to be sufficient to prevent oxidative damage.

**Methodology/Principal Findings:**

OXR1 has a complex genomic structure in the mosquito *A. gambiae*, and we confirm that multiple splice forms are expressed in adult females. Our studies revealed that OXR1 regulates the basal levels of catalase (CAT) and glutathione peroxidase (Gpx) expression, two enzymes involved in detoxification of hydrogen peroxide, giving new insight into the mechanism of action of OXR1. Gene silencing experiments indicate that the Jun Kinase (JNK) gene acts upstream of OXR1 and also regulates expression of CAT and GPx. Both OXR1 and JNK genes are required for adult female mosquitoes to survive chronic oxidative stress. OXR1 silencing decreases *P. berghei* oocyst formation. Unexpectedly, JNK silencing has the opposite effect and enhances *Plasmodium* infection in the mosquito, suggesting that JNK may also mediate some, yet to be defined, antiparasitic response.

**Conclusion:**

The JNK pathway regulates OXR1 expression and OXR1, in turn, regulates expression of enzymes that detoxify reactive oxygen species (ROS) in *Anopheles gambiae*. OXR1 silencing decreases *Plasmodium* infection in the mosquito, while JNK silencing has the opposite effect and enhances infection.

## Introduction

Reactive Oxygen Species (ROS) are highly reactive oxygen-derived molecules that include free radicals and peroxides. They are formed, in part, as natural byproducts of mitochondrial respiration and it is estimated that as much as 0.1 to 5% of all oxygen consumed by mitochondria is converted into ROS [1]. High levels of ROS can also be generated abruptly, as part of the immune response to pathogens [2], and the intracellular redox state is a key determinant of cell survival, proliferation, differentiation, and apoptosis [3].

ROS produced in living organisms have the potential to damage key cellular components including lipids, proteins and DNA. ROS-mediated DNA damage contributes to spontaneous mutagenesis that can lead to various functional disorders, including premature aging and cancer [4]. Cells protect themselves from ROS by preventing cell damage through detoxification of these chemicals, and by repairing ROS-induced damage once it takes place. For example, superoxide dismutases (SODs), convert superoxide anion into hydrogen peroxide, a less reactive species, while catalase detoxifies hydrogen peroxide into water and oxygen. In mammals, the nuclear factor kappa B (NF-κB) is actively involved in the induction of catalase and glutathione peroxidase (Gpx) expression in response to oxidative stress [5].

ROS act as signaling molecules and a number of transcription factors that regulate expression of antioxidant genes in response to ROS have been characterized in prokaryotes and in yeast [6] [7]. Hydrogen peroxide (H_2_O_2_) is one of the most stable and diffusible ROS signaling molecule and has been shown to activate the Jun N-terminal Kinase (JNK) [8] [9] and NF-κB [8] [5] signaling cascades. In *Drosophila*, the Jun N-terminal Kinase (JNK) pathway activates the transcription factor Activator Protein-1 (AP-1) and induces expression of stress-response genes [10].

The Oxidation Resistance 1 (OXR1) gene is present in all eukaryote genomes sequenced so far and is known to protect yeast and human cells from oxidative damage, through an unknown mechanism [11]. When human OXR1 is expressed in *Escherichia coli* mutants that are unable to repair oxidative damage, it has a protective effect, reducing the accumulation of mutations in the bacterial genome [12]. The OXR1 protein is localized in the mitochondria in yeast and human cells [12] [11], but nuclear localization has also been reported in mouse and rat cell lines [13] [14]. The most highly conserved region of the gene is the carboxyl-terminal TLDc domain, which has been shown to be sufficient to reduce the mutation rate in *E. coli* [15]. The TLDc domain is restricted to eukaryotes, and is commonly found in proteins that also contain lysin motifs (LysM) (IPR002482).

In *Drosophila melanogaster*, the OXR1 gene (CG32464) has 19 different transcripts that are generated by alternative splicing of 25 different exons (Entrez Gene and FlyBase). Eighteen of these transcripts share a common 195 aa C-terminal region (exons 24 and 25) that codes for the TLDc domain. This conserved region can splice with at least 6 different exons. The short isoforms splice a unique exon (either exon 19, 20, 21, 22 or 23) to exons 24–25, giving rise to five different OXR1 transcripts (isoforms A, C, H, I and Q). Twelve longer transcripts share exons 14–18 which are also spliced to exons 24–25 (E18 group) and only differ in the exon they splice at the 5′ of the mRNA. Genetic studies revealed that OXR1 (L82 gene) is a late puff gene with an essential developmental role. Deletion of this locus results in lethality due to the inability of adult flies to eclose [16]. OXR1 was also identified in a recent genetic screen for genes that affect the survival of *Plasmodium gallinaceum* parasites injected into the *Drosophila* hemocele [17]. Furthermore, OXR1 silencing reduces *Plasmodium berghei* infection in the mosquito *A. gambiae* [17]. In this study, we examined the participation of *A. gambiae* OXR1 in protecting mosquitoes from oxidative stress and on the survival of early stages of *P. berghei* parasites in the mosquito. The role of Jun Kinase (JNK) in the regulating the expression of OXR1 and ROS detoxification enzymes, and in limiting *Plasmodium* infection was also explored.

## Results

### OXR1 gene structure and phylogenetic analysis

**Figure 1:**
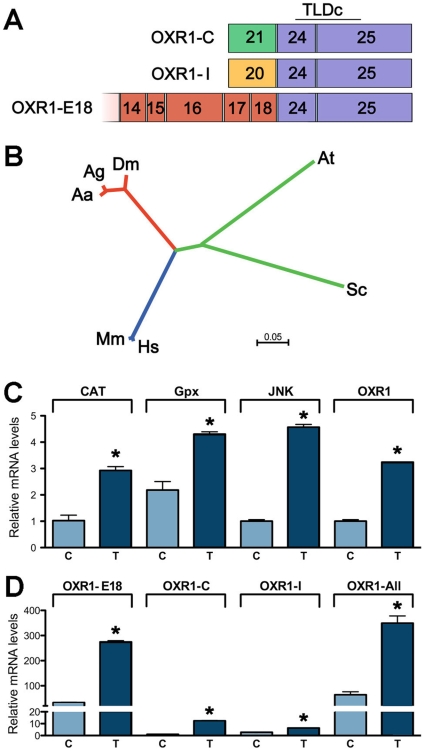
Genomic organization, phylogeny, and expression of the *An. gambiae* OXR1 gene. (A) Genomic organization of AgOXR1 gene and splice forms transcript B, C, and I. (B) Phylogenetic tree based on the sequence alignment of the deduced amino acid sequence of members of the OXR1 family from different species. (Hs  =  *Homo sapiens*, Mm =  *Mus musculus*, Dm  =  *Drosophila melanogaster*, Ae  =  *Aedes aegypti*, Ag  =  *Anopheles gambiae*, At  =  *Arabidopsis thaliana* and Sc  =  *Saccharomyces cerevisiae*). The sequence alignment used to build the dendrogram is shown in Figure S1. (C) Changes in Catalase, Glutathione peroxidase, JNK and OXR1 (TLDc domain region) mRNA levels 6 h after H_2_O_2_ injection (T) compared with water-injected control mosquitoes (C) in A. gambiae. Values were normalized using *An. gambiae* ribosomal protein S7 mRNA levels as an internal reference. Mean ± SEM. (D) Changes in mRNAs levels of specific OXR-1 isoforms (OXR1-C, OXR1-I and OXR1-E18) and of all OXR1 transcripts (OXR1-all, in which primers from the shared TLDc domain were used) were determined 6 h after injection of H_2_O_2_ (T  =  treated group) or water control (C  =  control group) in *An. gambiae* (G3 strain). Values were normalized using *An. gambiae* ribosomal protein S7 mRNA levels as an internal reference. Mean ± SEM. * indicates significant differences (p<0.05) by ANOVA.

The *A. gambiae* OXR1 gene (AGAP001751-PA) has a complex structure, consisting of at least 17 exons that span a 45 kb region. Bioinformatic analysis, based on the assembly of available ESTs and Blast analysis of all *Drosophila* exons against the *A. gambiae* genome sequence, identified several putative OXR1 exons in *A. gambiae.* We confirmed, by PCR amplification with exon-specific primers, that at least three different types of OXR1 mRNA transcripts are expressed in adult mosquito females (Fig. 1A): those in which exon 24 (numbering based on the DmOxR1 gene) splices with exon 21 (homolog of *Drosophila* isoform C), with exon 20 (homolog of *Drosophila* isoform I), or with exon 18 (E18 group).

The deduced amino acid sequence of the TLDc domain of AgOXR1 was aligned with that of *Aedes aegypti* and *Culex quinquefasciatus* mosquitoes, *Drosophila melanogaster*, Human, Mouse, Arabidopsis and Yeast (Fig. S1) and their phylogeny was analyzed (Fig. 1B). The AgOXR1 deduced amino acid sequence is most similar to that of its putative orthologs in *A. aegypti* and *D. melanogaster* (96% and 87% identity, respectively), and has diverged extensively from that of mammals, plants and yeast (52%, 36% and 28% identity, respectively); indicating that OXR1 is an ancient gene that was probably already present in the common ancestor of yeast and humans.

### Oxidative stress induces expression of multiple OXR1 isoforms

To examine the involvement of OXR1 in the resistance of *A. gambiae* to oxidative stress, adult mosquito females were challenged by injecting H_2_O_2_ directly into the hemocele. As previously shown [18], this challenge induced expression of Catalase and Glutathione peroxidase (Gpx), two enzymes that detoxify H_2_O_2_, by 3 and 2 fold, respectively, relative to the water-injected control group (P = 0.018 and P = 0.024) (Fig. 1C). Expression of all OXR1 forms (using primers from the TLDc domain) and JNK also increases by 3 fold (P = 0.01 and P = 0.001, respectively); indicating oxidative stress also activates transcription of these two genes. Interestingly, OXR1 mRNA levels do not change significantly in response to a bacterial challenge with a mixture of *E. coli* and *Micrococcus luteus* (Fig. S2).

The individual response of the three types of OXR1 transcripts to oxidative stress was also analyzed in adult females. The OXR1-exon18 group is the predominant form present in adult females (Fig. 1D) and expression of all AgOXR1 isoforms (Isoforms I, C and the Exon18 group) increases significantly in response to H_2_O_2_ injection (P<0.01) (Fig.1D). In all subsequent experiments, primers to quantitate OXR1 mRNA expression levels and dsRNA constructs to silence gene expression were designed from the TLDc domain, which is common to all OXR1 isoforms.

### OXR1 regulates expression of ROS detoxification enzymes through the Jun kinase (JNK) pathway

**Figure 2:**
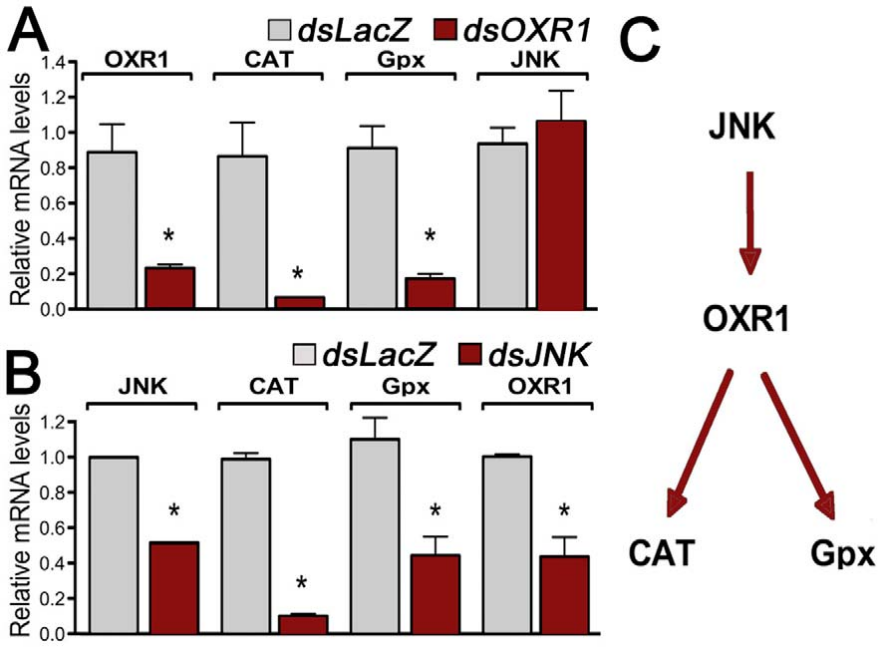
Effect of OXR1 silencing on JNK and ROS detoxification enzymes mRNA levels and effect of JNK silencing on OXR1 and ROS detoxification enzymes mRNA levels. (A) Changes in OXR1, Catalase, Glutathione peroxidase, JNK mRNA levels in *dsOXR1* injected mosquitoes compared to *dsLacZ* control injected mosquitoes. (B) Changes in JNK, Catalase, Glutathione peroxidase, OXR1 mRNA levels in *dsJNK*-injected mosquitoes compared to *dsLacZ* control injected mosquitoes All transcript measurements were performed using qRT-PCR. Data are shown as Mean + SE. * indicates significant differences (p<0.05) by ANOVA.(C) Diagram of the proposed organization of the OXR1 signaling cascade in A. gambiae adult females.

In spite of the well-defined role of OXR1 in protection against ROS damage in several organisms, the mechanism of this protective effect has not been established. The hypothesis that OXR1 regulates expression of ROS detoxification enzymes in *A. gambiae* was investigated. OXR1 silencing decreases endogenous Catalase and Gpx mRNAs by 92% and 82% respectively (p<0.001) but does not affect JNK mRNA levels, relative to the *dsLacZ* group (Fig. 2A). OXR1 silencing also decreases expression of SOD1, SOD2 and SOD3 but, in general, the effect is less striking (38%, 50% and 46% reduction, respectively, p<0,01) (Fig. S3).

In *Drosophila*, the JNK pathway has been shown to activate expression of stress-response genes [10]. We investigated whether JNK could be involved in regulating expression of OXR1, Catalase or Gpx in *A. gambiae*. Injection of dsJNK RNA reduced JNK mRNA levels by 50% relative to the ds*LacZ* control. Furthermore, JNK silencing significantly decreases Catalase, Gpx and OXR1 mRNA levels by 90%, 60% and 56%, respectively p<0.0001) (Fig. 2B). Taken together, these gene-silencing experiments indicate that JNK and OXR1 regulate the basal mRNA levels of ROS detoxification enzymes and that JNK acts upstream of OXR1 (Fig. 2C).

### OXR1 and JNK are essential for mosquitoes to survive chronic oxidative stress

**Figure 3:**
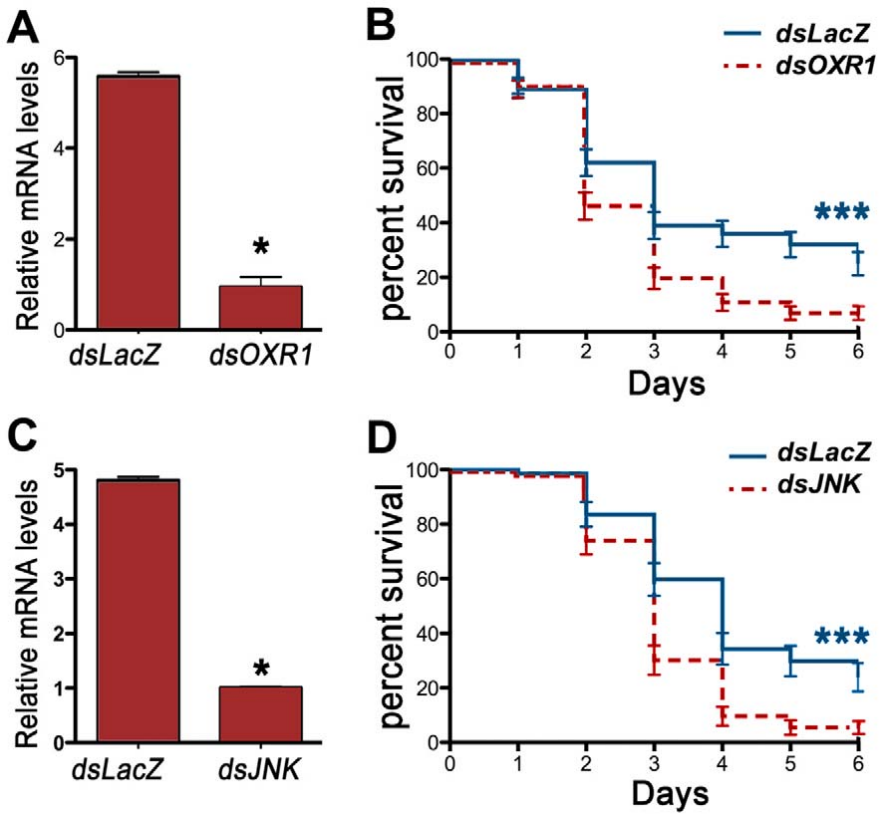
Effect of OXR1 midgut silencing on *A. gambiae* G3 adult females survival when challenged by H_2_O_2_ chronic oral administration. (A) Relative amounts of midgut OXR1 mRNA after injection with *dsOXR1* compared with *dsLacZ* injected control mosquitoes. (B) Relative amounts of midgut JNK mRNA after injection with *dsJNK* compared with *dsLacZ* injected control mosquitoes. (C) % Survival of control *dsLacZ* and *dsOXR1*-injected mosquitoes after 1% H_2_O_2_ chronic feeding over 6 days. (D) % Survival of control *dsLacZ* and *dsJNK*-injected mosquitoes after 1% H_2_O_2_ chronic feeding over 6 days. Significance was determined by Logrank test. * * * significance(p<0.001).

To determine whether OXR1 and JNK are required for mosquitoes to withstand chronic oxidative stress, groups of 3 day-old females were injected with dsLacZ, dsOXR1 or dsJNK and the efficiency of midgut silencing was tested 36 h post-infection (hpi). Endogenous levels of midgut OXR1 and JNK mRNA were reduced by 83 and 80%, respectively, relative to the dsLacZ control group (Fig. 3A and 3C). The effect of feeding mosquitoes water containing 1% H_2_O_2_ on daily survival was evaluated in triplicate groups for each treatment. This high dose of hydrogen peroxide caused substantial mortality in all experimental groups, but the mortality was significantly higher (P<0.0001, Log rank test) when either dsOXR1 (Fig. 3B) or dsJNK (Fig. 3D) were silenced. These experiments indicate that OXR1 and JNK play a critical role in protecting adult females mosquitoes from chronic oxidative stress.

### OXR1 silencing limits *Plasmodium* oocyst formation in *A. gambiae*

**Figure 4:**
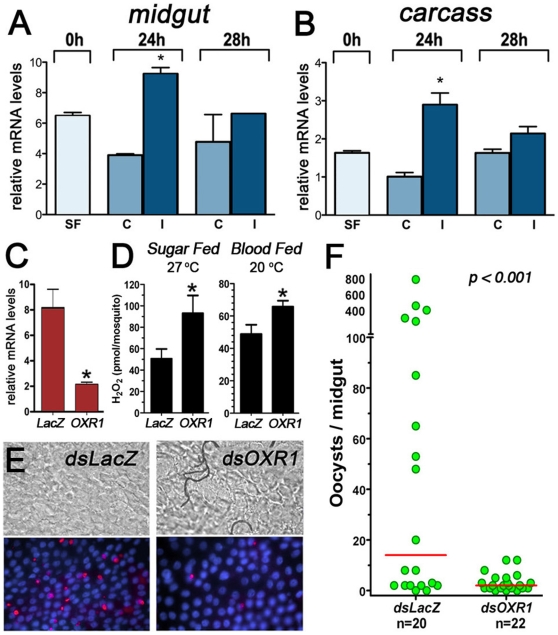
AgOXR1 expression in response to *P. berghei* infection and effect of AgOXR1 silencing on parasite survival in *Plasmodium*-infected *An. gambiae* (G3 Strain) females. (A) Changes in OXR1 mRNA levels in response to P. berghei infection (0, 24, 28 h hr PI) in midgut samples. (B) Changes in OXR1 mRNA levels in response to *P. berghei* infection (0, 24, 28 h hr PI) in carcass samples (whole body without the midgut). (C) Effect of dsOXR1 injection on OXR1 mRNA levels in G3 mosquitoes relative to a control group injected with LacZ dsRNA (*dsLacZ*). All transcript determinations were performed using qRT-PCR. (D) Effect of OXR1 silencing on H_2_O_2_ hemolymph levels of individual female mosquitoes (n = 10) that were either sugar-fed and kept at 27°C (left panel) or blood-fed on a healthy mouse and kept at 20°C (right panel), the temperature at which *Plasmodium*-infected mosquitoes are kept. Data are shown as Mean + SE. The asterisk indicates significant differences (p<0.05) by ANOVA. (E–F) Effect of dsRNA-mediated knockdown of *An. gambiae* OXR1 in *P. berghei* midgut infection after 48 h post-infection in *dsOXR1* or *dsLacZ* injected mosquitoes. (D) Representative fields of immunofluorescence stainings of early *P. berghei* oocysts 48 h post-infection. Nuclei are in blue and *P. berghei* oocysts are in red. Scale bar 10 µm. (E) The dots represent the number of parasites present on individual midguts and the median number of oocysts is indicated by the horizontal line. The distributions are compared using the Kolmogorov-Smirnov test (KS test), n  =  number of mosquitoes, * significantly different with respect to *dsLacZ* injected controls (Kolgomorof-Smirnoff test, p<0.05).

We have previously shown that OXR1 silencing decreases the number of *Plasmodium berghei* mature oocysts present six days post-infection (dpi) [17]. To better define the stage of the parasite that is affected by OXR1 silencing, we analyzed OXR1 expression in the midgut and carcass (rest of the mosquito without midgut) at early stages of infection, 24 and 28 h hpi. We found that *Plasmodium* infection induces OXR1 mRNA expression in the midgut and systemically 24 hpi, at the time when ookinetes are invading the midgut epithelium (Fig. 4A). This transcriptional response is transient, as it is no longer observed by 28 hpi (Fig. 4B). To investigate whether OXR1 silencing reduces the number of *Plasmodium* ookinetes that complete invasion and transform into oocysts, early oocyst formation was analyzed two days PI using immunofluorescence staining. Injection of dsOXR1 decreases OXR1 mRNA levels by 78%, relative to the dsLacZ control (Fig. 4C) and results in a significant increase in hemolymph H_2_O_2_ levels both in sugar-fed females (kept at 27°C) and in blood-fed females 24 hpi (Fig. 4D). OXR1 silencing also reduces the median number of early oocysts present 2dpi by 7 fold, relative to the dsLacZ-injected control (p<0.001, KS test) (Fig. 4 E–F).

### JNK silencing enhances *Plasmodium* infection

**Figure 5:**
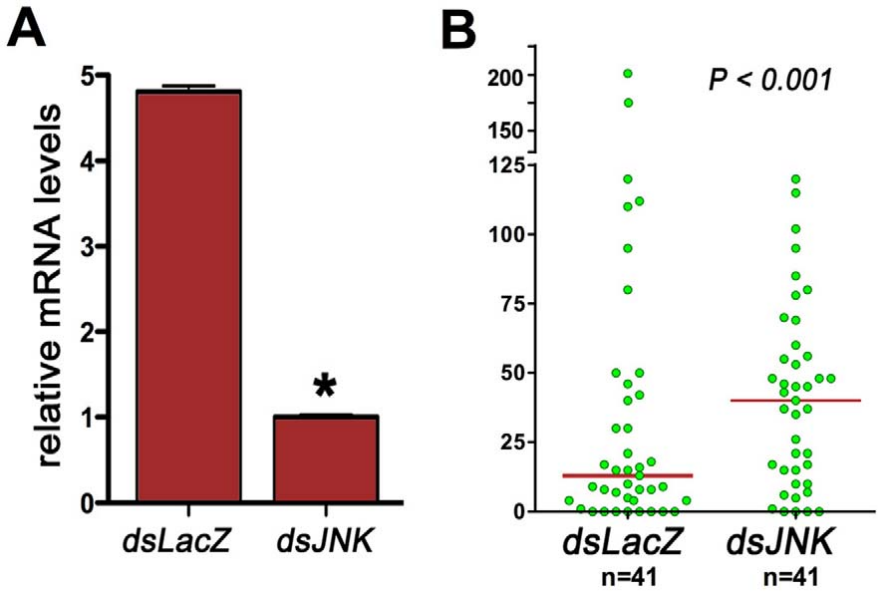
Effect of AgOXR1 silencing on parasite survival in *Plasmodium*-infected *A. gambiae* (G3 Strain) females. (A) Relative amounts of JNK mRNA in G3 mosquitoes after injection with JNK dsRNA (dsJNK) compared with controls injected with LacZ dsRNA (*dsLacZ*). (B) Effect of dsRNA-mediated knockdown of *An. gambiae* JNK in *P. berghei* midgut infection after 6 days post-infection in *dsJNK* or *dsLacZ* injected mosquitoes. The dots represent the number of parasites present on individual midguts and the median number of oocysts is indicated by the horizontal line. The distributions are compared using the Kolmogorov-Smirnov test (KS test), n  =  number of mosquitoes, * significantly different with respect to *dsLacZ* injected controls (Kolgomorof-Smirnoff test, p<0.05).

As JNK silencing reduces OXR1 expression (Fig. 3B), we hypothesized that JNK silencing would have a similar effect as OXR1 depletion, and would decrease *Plasmodium* infection (Fig. 2C). JNK silencing reduced whole body mRNA levels by 82% (Fig. 5A). However, this reduction in JNK expression has the opposite effect as OXR1 silencing, increasing the mean number of oocysts present 6 days PI by three fold (P<0.001) (Fig. 5B).

## Discussion

ROS are critical for *A. gambiae* to mount effective immune responses against bacteria and *Plasmodium* [18]. Previous studies showed that a mosquito line selected to be refractory (R) to *Plasmodium* infection has higher systemic levels of H_2_O_2_ than unselected (G3) or susceptible (S) females [19]. High H_2_O_2_ levels in the R strain are also associated with increased survival to bacterial injection. Conversely, mortality is higher in the S strain, which has the lowest level of H_2_O_2_. Reduction of ROS by dietary supplementation of Vitamin C or Uric Acid (strong antioxidants), dramatically decreases survival following a bacterial challenge [18].

We have previously shown that catalase silencing results in higher systemic levels of H_2_O_2_, increased ookinete lysis and reduced oocyst formation [18]. Because OXR1 silencing reduces catalase expression (Fig. 2A) and increases systemic levels of H_2_O_2_ (Fig. 4D), it is not surprising that it also decreases *P. berghei* infection. However, the unexpected finding that JNK silencing has the opposite effect on *Plasmodium* and enhances infection suggests that, besides its role in ROS balance, this cascade may also mediate an antiparasitic response, yet to be defined. In *Drosophila*, tissue damage activates the JNK signaling, which induces expression of cytokines that activate the STAT pathway [20]. In *A. gambiae*, the STAT pathway mediates antiplasmodial responses that target the oocysts stage by activating expression of nitric oxide synthase [21]. It is thus possible that JNK signaling participates in the activation of the STAT pathway during the late-phase antiparasitic response in *A. gambiae*.

A recent study revealed that *P. berghei* parasites are particularly vulnerable to oxidative stress in mosquito stages of their life cycle; as disruption of the glutamylcysteine synthetase (c-GCS) gene, the first enzyme in the glutathione biosynthesis pathway, has no effect in asexual stages, but results in stunted oocysts unable to produce infective sporozoites [22]. Different species (or strains) of *Plasmodium* may also differ in their sensitivity to oxidative stress. For example, OXR1 depletion in *A. gambiae* does not affect infection with the African *Plasmodium falciparum* 3D7 strain [23]. This raises the question of whether *P. falciparum* is less sensitive to ROS than *P. berghei* or whether it generates less ROS. Previous expression analysis revealed that 28 genes linked to mitochondrial, oxido-reductive or stress responses are induced in *A. gambiae* midguts infected with *P. berghei*, while only 14 genes are induced in *P. falciparum*-infected midguts [24]; suggesting that infection with *P. falciparum*, a well-adapted system, may be less stressful to the mosquito.

Although OXR1 has been shown to confer resistance against oxidative stress in several eukaryote organisms [12] [11] [14], the mechanism mediating this protective effect has not been elucidated. Our studies confirmed that OXR1 is also important to protect mosquitoes from oxidative stress and revealed that OXR1 mRNA levels are induced in response to systemic injection of hydrogen peroxide. Furthermore, we found that OXR1 regulates the basal levels of catalase and Gpx expression, two protective enzymes involved in the detoxification of hydrogen peroxide, providing new insight into the mechanism of action of OXR1. Previous studies showed that the JNK pathway regulates expression of stress-response genes in *Drosophila*, such as heat shock proteins, and that it is critical for flies to tolerate to chronic oxidative stress [10]. Our data provide direct experimental evidence that JNK acts upstream and regulates OXR1 expression. One can envision a regulatory cascade in which high systemic levels of hydrogen peroxide activate JNK signaling, which in turn activates transcription factors by phosphorylating them (probably AP-1 transcription factor complex, consisting of a Jun and Fos dimer) which, in *Drosophila*, are known to activate expression of JNK-target genes [10]. Our data suggest that both the JNK and OXR1 genes are potential targets genes of this cascade. Our studies indicated that OXR1 also regulates expression of Catalase and Gpx, but the molecular mechanism of actions of OXR1 remains to be defined. The role of OXR1 could be a direct one, as a cofactor that modulates transcriptional activation of Catalase and Gpx. If this were the case, one would expect the OXR1 protein to have a nuclear localization. Alternatively, the effect of OXR1 on Catalase and Gpx expression could be and indirect one. In some studies the OXR1 protein has been localized to the mitochondria, suggesting that OXR1 could also modulate ROS generation or signaling by this organelle. OXR1 is a complex gene that is expressed as multiple splice forms. It is possible that the different OXR1 isoforms localize to different subcellular compartments and carry out diverse functions. Silencing expression of specific variants and generating antibodies to isoform-specific exons would be required understand the functional significance of this complex differential splicing. We conclude that both JNK and OXR1 are critical components of the ROS-response system in *A. gambiae* and that one of the mechanism by which they protect mosquitos from oxidative damage is by regulating expression of Catalase and GPX, and thus modulating their ability to detoxify ROS.

### Experimental procedures

Ethics Statement: Animal care guidelines were followed according to the NIH Animal Care and Use Committee (ACUC).

### Sequence and phylogenetic analysis

The predicted amino acid sequence of several members of the OXR1 family from different species (Ms = *Mus musculus* Hs  =  *Homo sapiens*, Dm  =  *Drosophila melanogaster*, Ae  =  *Aedes aegypti*, Ag  =  *Anopheles gambiae*, At  =  *Arabidopsis thaliana* and Sc  =  *Saccharomyces cerevisiae*) were aligned and dendrograms constructed using the ClustalW software (Thompson *et al.*, 1994) and the results were visualized graphically with TreeviewX 0.5.0. The sequence alignment appears in Figure S1.

### *P. berghei* Infection of Mosquitoes

Female mosquitoes (5 days old) were infected with *P. berghei* by feeding on anesthetized infected BALB/c mice. Mosquitoes were fed when mice reached 4–10% parasitemia and had at least 1 exflagellation per field, following standard procedures previously described [25]. Blood-fed infected and control mosquitoes were kept at 21°C and 80% humidity. *P. berghei* midgut infection was quantified 48 h post-infection by immunofluorescence using mouse anti Pbs21 antibody as previously described [26].

### Quantitation of Gene Expression

*A. gambiae* midguts/carcasses/whole bodies were collected from 10 unfed or blood-fed females. All samples were collected in duplicate groups, frozen in liquid nitrogen and stored at −80°C. Poly(A) mRNA or total RNA was isolated using Oligotex-dT beads (Qiagen) or RNAeasy (Qiagen) respectively, following the manufacturer's instructions. First-strand cDNA was synthesized using QuantiTect reverse transcriptase (Qiagen). Gene expression was assessed by SYBR green quantitative real-time PCR (qPCR) (DyNAmo HS; New England Biolabs) in a Chromo4 system (Bio-Rad). PCR involved an initial denaturation at 95°C for 15 min, 44 cycles of 10 sec at 94°C, 20 sec at 58°C, and 30 sec at 72°C. Fluorescence readings were taken at 72°C after each cycle. A final extension at 72°C for 5 min was completed before deriving a melting curve (70–95°C) to confirm the identity of the PCR product. qRT-PCR measurements were made in duplicate. Primers used for qPCR amplify an OXR1 fragment nonoverlapping with the fragment used for RNAi. Relative quantitation results were normalized with *A. gambiae* ribosomal protein S7 as internal standard and analyzed by the 2^–ΔΔ^Ct method [27]. Primers used are provided in Table S1.

### Bacterial Challenge of Mosquitoes

Three-day-old adult females were injected with a mixture of *Escherichia coli* and *Micrococcus luteus*. Bacterial cultures were grown to an optical density of 0.5 (600 nm) in LB broth, and 200 µl from each culture were mixed and centrifuged for 5 min at maximum speed. The supernatant was discarded, and the pellet was washed twice with PBS and resuspended in 125 µl of PBS. Mosquito survival was monitored daily for 8 days after intrathoracic injection of 0.138 µl of either PBS or bacterial suspension using a microinjection system and a micromanipulator (Nanoject II microinjection system; Drummond).

### H_2_O_2_ Injection of Mosquitoes

Sugar-fed 4-day-old *A. gambiae* G3 females were injected as above with 18 nmol of H_2_O_2_ (in 69 nl); controls were injected with water. Mosquitoes were kept at 27°C, and 6 h after treatment, groups of 10 were collected as previously described [18].

### Cloning and sequencing of OXR1 C-terminal domain

Adult females were fed on a healthy mouse and collected 24 h after blood feeding. Whole mosquitoes were collected and stored at –70°C until mRNA extraction. Poly(A) mRNA was isolated from groups of 10 whole mosquitoes using Oligotex-dT beads (Qiagen), following the manufacturer's instructions. First-strand cDNA was synthesized using random hexamers and Superscript II reverse transcriptase (Invitrogen). The AgOXR1 C-terminal cDNA fragment was cloned by using the cDNA obtained from the 10 whole mosquitoes as template.

All OXR1 sequences available in EST databases were aligned using the Sequencher 4.8 (Mac) program. Primers were designed to amplify the conserved C-terminal domain (exons 24–25 in Fig. 1A) and the PCR product was cloned and sequenced. The nucleotide and deduced amino acid sequences are appear in Figure S3.

### RNAi gene-silencing assays

Sense and antisense RNAs were synthesized from PCR-amplified gene fragments using the T7 Megascript kit (Ambion). dsRNA was further purified with water and concentrated to 3 µg/µl using a Microcon YM-100 filter (Millipore). The sequences of the primers are listed in Table S2. The gene specific amplicon is cloned into the pCR_IITOPO vector (Invitrogen). T7 polymerase promoter sites were incorporated onto both ends of this fragment by amplification with the following vector primers: forward 5′-CTCGAGTAATACGACTCACTATAGGGCTAGTAACGGCCGCCAGTGT-3′ and reverse 5′-CTCGAGTAATACGACTCACTATAGGGGCCAGTGTGATGGATATCTGC-3′. The PCR product was used as template to synthesize double-stranded of the target gene RNA *in vitro* using the MEGAscript RNA-mediated interference kit (Ambion). About 69 nl of dsRNAs (207 ng of dsRNA) in water was introduced into the thorax of cold-anesthetized 1-day old (for systemic silencing) or 3–4 days-old (midgut silencing) female mosquitoes using a nano-injector (Nanoject; Drummond Scientific, Broomall, Pennsylvania,United States) with a glass capillary needle according to established methodology [28]. For gene-silencing assays, 1-day-old or 3-day old female mosquitoes were injected, in parallel, with LacZ dsRNA as a control group or with target gene–specific dsRNA for the experimental group. Gene silencing was verified 24 h after dsRNA injection by real-time quantitative RT-PCR, with the *A. gambiae* ribosomal S7 gene as the internal control for normalization. The primers for silencing verification are listed in Table S1.

### H_2_O_2_ chronic feeding of Mosquitoes

Adult females fed on a 10% sugar solution were injected with dsRNA at 3 days post emergence and fed overnight on the same 10% sugar solution. On day 4 PE they were fed on a sugar cube and water separately, to ensure that the crop was empty. On day 5 PE the treatment to induce chronic oxidative stress was initiated by feeding them on a 10% sugar solution containing 1% H_2_O_2_ until they died. Mortality was recorded over a period of 6 days. Two groups of 50 mosquitoes were used for each treatment. Significance was determined by Log rank test (p<0.005).

### Hemolymph H_2_O_2_ quantitation

Mosquito hemolymph was collected by flushing the hemocoel. The cuticle from the last abdominal segment was torn open, 8 µl of PBS containing 2 mg/ml of catalase inhibitor (3-amino-triazole) were injected into the thorax and 5 µl of flushed hemolymph collected from the aperture in the posterior end. Levels of H_2_O_2_ in the hemolymph of 10 individual mosquitoes was determined using the Amplex red assay fluorimetric test (Molecular probes) for each experimental treatment, following the manufacturer instructions. The results are shown as the Mean ± SE of each experimental treatment.

## Supporting Information

Figure S1 Sequence alignment of the TLDc Domain of the OXR1 genes from different species (Hs  =  *Homo sapiens*, Mm =  *Mus musculus*, Dm  =  *Drosophila melanogaster*, Ae  =  *Aedes aegypti*, Ag  =  *Anopheles gambiae*, At  =  *Arabidopsis thaliana* and Sc  =  *Saccharomyces cerevisiae*).
(0.03 MB DOC)

Figure S2 OXR1 mRNA expression in sugar-fed adult females challenged with bacteria. OXR1 mRNA in whole body at different times after mosquitoes were challenged with a mixture of heat-killed *E. coli* and *M. luteus*. All transcript measurements were performed using qRT-PCR. Data are shown as Mean + SE. * indicates significant differences (p<0.05) by ANOVA.
(0.82 MB TIF)

Figure S3 Effect of OXR1 silencing on JNK and ROS detoxification enzymes mRNA induction upon blood feeding. Effect of OXR1 silencing on mRNA expression levels of (A) OXR1,(B) SOD1, (C) SOD2, (D) SOD3a in 24 h uninfected blood fed females (whole body) compared to *dsLacZ* control injected mosquitoes. All transcript measurements were performed using qRT-PCR. Data are shown as Mean + SE.
(1.42 MB TIF)

Table S1 Primers used to determine gene expression by qRT-PCR in *An. gambiae*.
(0.04 MB DOC)

Table S2 Primers used to produce PCR Amplicons for dsRNA Synthesis in *An. gambiae*.
(0.03 MB DOC)
