# Supplementary material for: The Anopheles gambiae Oxidation Resistance 1 (OXR1) Gene Regulates Expression of Enzymes That Detoxify Reactive Oxygen Species
Source: PLoS One. 2010 Jun 17;5(6):e11168. doi: 10.1371/journal.pone.0011168 (PMC2887368; doi:10.1371/journal.pone.0011168)
Supplement: Figure S1 — Sequence alignment of the TLDc Domain of the OXR1 genes from different species (Hs = Homo sapiens, Mm = Mus musculus, Dm = Drosophila melanogaster, Ae = Aedes aegypti, Ag = Anopheles gambiae, At = Arabidopsis thaliana and Sc = Saccharomyces cerevisiae). (0.03 MB DOC) [file pone.0011168.s001.doc]

Hs KQVATVKADLESESFRPNLSDPS-ELLLPDQIEKLTKHLPPRTIGY-PWTLVYGTGKHGT 58

Mm KQVAPAKADLEPESFRPNLSDPS-ELLLPDQIEKLTKHLPPRTIGY-PWTLVYGTGKHGT 58

Aa KATLFATSSFDQDFQIPDLIGQT-EILSEEHREKLCAHLPARAEGY-SWSLVFSTSLHGF 58

Cq KAALFATSSFDQDFQIPDLIGQT-EILSEEHREKLCAHLPARAEGY-SWSLVFSTSLHGF 58

Ag KATLFASGSFDQDFQIPDLVGQT-EILSEEHREKLCAHLPARAEGY-SWSLVFSTSQHGF 58

Dm KTSLFATGSFDLDFPIPDLIGKT-EILTEEHREKLCSHLPARAEGY-SWSLIFSTSQHGF 58

At NNNVKEEVTVVVQAIIPEISEPS-LLLSEQSRRSLYTSLPALVQGR-KWILLYSTWRHGI 58

Sc --TIVEEGSLPPVRLNGYLPSTKNKLLTPEMCDEIRTLMPTRIQLYTEWNLLYSLEQHGS 58

. : . :* : .: :*. * *::. **

Hs SLKTLYRTMTG-----LDTPVLMVIKDSDGQVFGALASEPLKVSDG--FYGTGETFVFTF 111

Mm SLKTLYRTMTG-----LDTPVLMVIKDSDGQVFGALASEPFKVSDG--FYGTGETFVFTF 111

Aa SLNSLYRKMHK-----LESPILIVIEDTEHNVFGALTSCSLHVSDH--FYGTGESLLYKF 111

Cq SLNSLYRKMHK-----LESPILIVIEDTDHNVFGALTSCSLHVSDH--FYGTGESLLYKF 111

Ag SLNSLYRKMHK-----LESPILIVIEDTDHNVFGALTSCSLHVSDH--FYGTGESLLYKF 111

Dm ALNSLYRKMAR-----LESPVLIVIEDTEHNVFGALTSCSLHVSDH--FYGTGESLLYKF 111

At SLSTLYRKSLL-----WPGLSLLVVGDRKGSVFGGLVEAPLIPTDKK-YQGTNSTFVFTN 112

Sc SLHSLYSNVAPDSKEFRRVGYVLVIKDRKNGIFGAYSNEAFHPNEHRQYTGNGECFLWKL 118

:* :** . ::*: * . :**. . .: .: : *... :::.

Hs CPE-----------------------------FEVFKWTGDNMFFIKGDMDSLAFGGGGG 142

Mm CPE-----------------------------FEVFKWTGDNMFFIKGDMDSLAFGGGGG 142

Aa NPH-----------------------------FKVFHWSGENLYFIKGNPESLAIGAGDG 142

Cq NPH-----------------------------FKVFHWSGENLYFIKGNPESLAIGAGDG 142

Ag NPH-----------------------------FKVFHWSGENLYFIKGNPESLAIGAGDG 142

Dm NPS-----------------------------FKVFHWTGENMYFIKGNMESLSIGAGDG 142

At KSG----------------------------QPTIYRPTGANRFYTLCSKEFLALGGG-G 143

Sc DKVPDVNISEKEESEQEGKEGKEEGDKEERWRFSGYPYTGVNEFAIYCTSEFLSMGAGDG 178

: :* * : : *::*.* *

Hs EFALWLDGDLYHGRSHSCKTFGNRTLSKK-EDFFIQDIEIWAFE---------------- 185

Mm EFALWLDGDLYHGRSHSCKTFGNHTLSKK-EDFFIQDIEIWAFE---------------- 185

Aa KFGLWLDGDLNQGRSQYCSTYSNEPLAPQ-EDFVIKTLECWAFV---------------- 185

Cq KFGLWLDGDLNQGRSQHCSTYSNEPLAPQ-EDFVIKTLECWAFV---------------- 185

Ag KFGLWLDGDLNQGRSQHCSTYSNEPLAPQ-EDFVIKTLECWAFV---------------- 185

Dm RFGLWLDGDLNQGRSQQCSTYGNEPLAPQ-EDFVIKTLECWAFV---------------- 185

At RFALYLDSELLSGSSAYSETYGNSCLADS-QDFDVKEVELWGFV---------------- 186

Sc HYGLLCDDGLLHGVSNPCQTYGNEVLSKEGKKFSIVALEVWRVG---------------- 222

.:.* *. * * * ..*:.* *: . :.* : :* * .
