# Supplementary figures and images for: The Anopheles gambiae Oxidation Resistance 1 (OXR1) Gene Regulates Expression of Enzymes That Detoxify Reactive Oxygen Species
Source: PLoS One. 2010 Jun 17;5(6):e11168. doi: 10.1371/journal.pone.0011168 (PMC2887368; doi:10.1371/journal.pone.0011168)

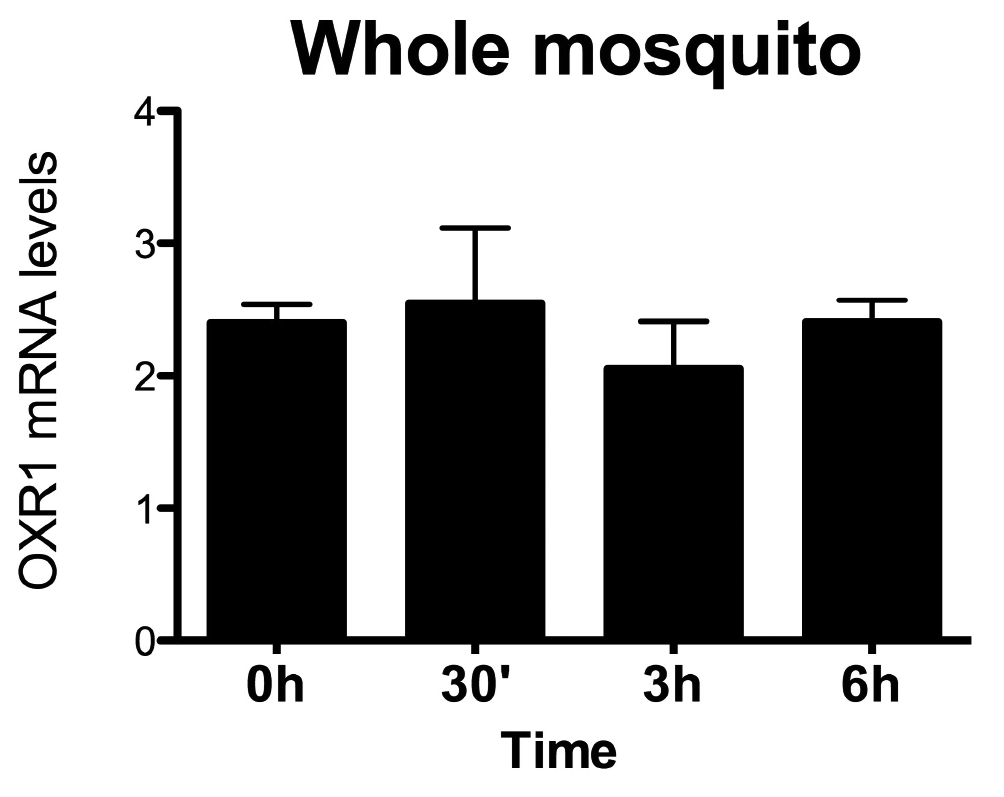

Supplement: Figure S2 — OXR1 mRNA expression in sugar-fed adult females challenged with bacteria. OXR1 mRNA in whole body at different times after mosquitoes were challenged with a mixture of heat-killed E. coli and M. luteus. All transcript measurements were performed using qRT-PCR. Data are shown as Mean + SE. * indicates significant differences (p<0.05) by ANOVA. (0.82 MB TIF) [file pone.0011168.s002.tif]

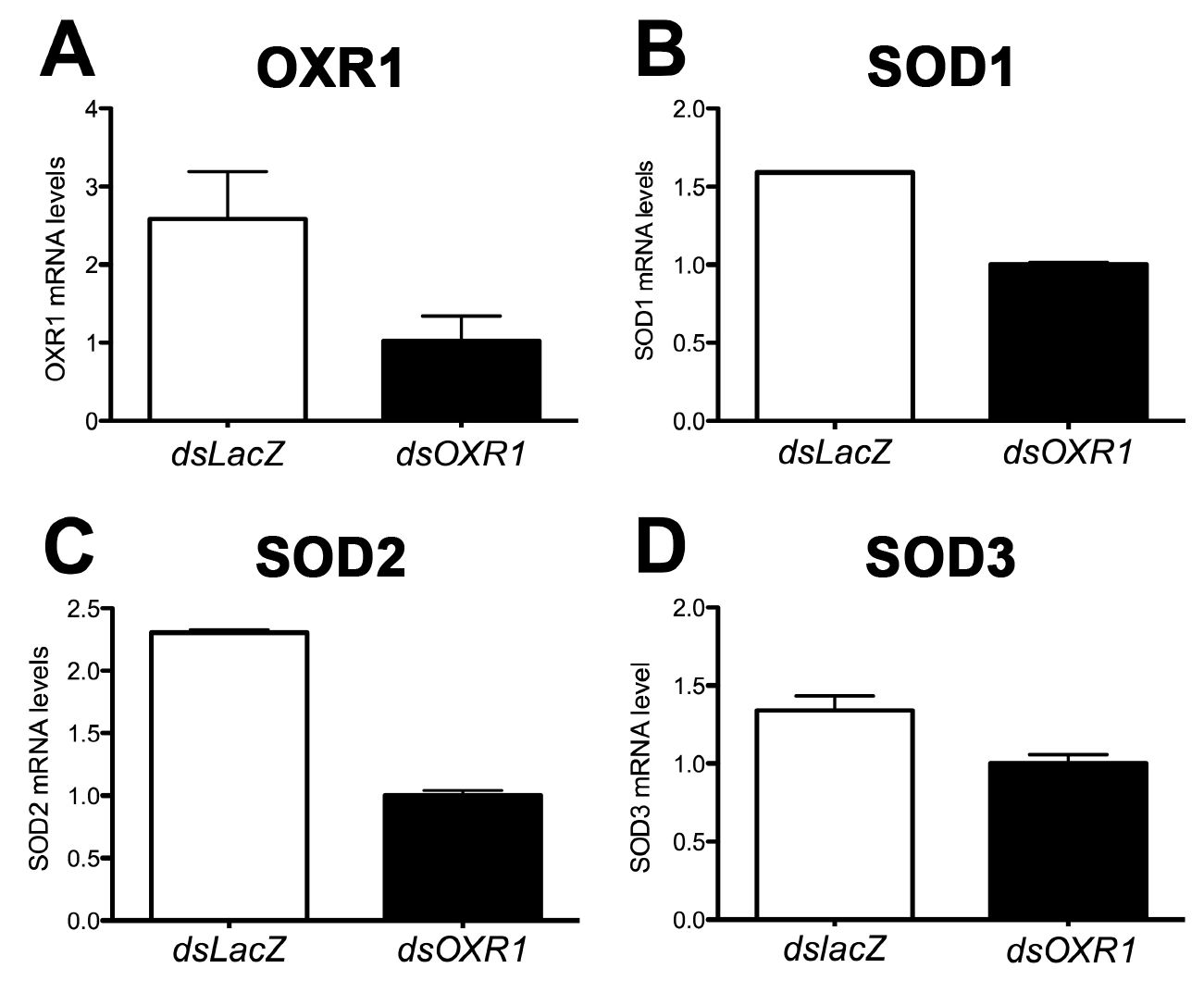

Supplement: Figure S3 — Effect of OXR1 silencing on JNK and ROS detoxification enzymes mRNA induction upon blood feeding. Effect of OXR1 silencing on mRNA expression levels of (A) OXR1,(B) SOD1, (C) SOD2, (D) SOD3a in 24 h uninfected blood fed females (whole body) compared to dsLacZ control injected mosquitoes. All transcript measurements were performed using qRT-PCR. Data are shown as Mean + SE. (1.42 MB TIF) [file pone.0011168.s003.tif]
