# Supplementary material for: The Anopheles gambiae Oxidation Resistance 1 (OXR1) Gene Regulates Expression of Enzymes That Detoxify Reactive Oxygen Species
Source: PLoS One. 2010 Jun 17;5(6):e11168. doi: 10.1371/journal.pone.0011168 (PMC2887368; doi:10.1371/journal.pone.0011168)
Supplement: Table S1 — Primers used to determine gene expression by qRT-PCR in An. gambiae. (0.04 MB DOC) [file pone.0011168.s004.doc]

| **Gene** | **Primer sequence** |
| --- | --- |
| OXR1  TLDc | Fw 5’-GCACAGATGACTACAGAAAAGCGAC-3’  Rv 5’-AACACGAGAGACCACGAGTATCCC-3’ |
| OXR1  Iso E18 | Fw 5’-GCATCGATATCCGTGACCC-3’  Rv 5’- AAGAAGACGTCCAGCGTGAG-3’ |
| OXR1  Iso I | Fw 5’- GTAAGCTGGTTCGGGGTTTT-3’  Rv 5’-CGTTTTCGCTAACGGCACT-3’ |
| OXR1  Iso C | Fw 5’-CCTGTACTTCGCCTCGAAAC -3’  Rv 5’- GAACGGACGTATCGATCAGC-3’ |
| CAT | Fw 5'-GGATAAGGTGACGGCTACGA-3'  Rv 5'-GAGAAGCGTACGGCTAGTGG-3' |
| Gpx | Fw 5'-CAAGGTGCTGCTAATCGTCA-3'  Rv 5'-CCTTATCCGCGTACTTCTGC-3' |
| JNK | Fw 5-TGCCAGGTCATACAGATGGA  Rv 5'-CCCAAAGTCGAGGATTTTCA |
| SOD1 | Fw 5'-GGAGCACTCTTTTCCACTGC-3'  Rv 5'-GGTGGTGAAGCTCCATGATT-3' |
| SOD2 | Fw 5'-CACATTCACGAGAAGGTGA-3'  Rv 5'-CGTGTCCGAGTAGGACGTTT-3' |
| SOD3a | Fw 5'-GGTAGACTTGACCGCTACGC-3'  Rv 5'-GCTCAGTTCATGTCCCCCTA-3' |
| S7 | Fw 5'-AGAACCAGCAGACCACCATC-3'  Rv 5'-GCTGCAAACTTCGGCTATTC-3' |
