# Supplementary material for: The Anopheles gambiae Oxidation Resistance 1 (OXR1) Gene Regulates Expression of Enzymes That Detoxify Reactive Oxygen Species
Source: PLoS One. 2010 Jun 17;5(6):e11168. doi: 10.1371/journal.pone.0011168 (PMC2887368; doi:10.1371/journal.pone.0011168)
Supplement: Table S2 — Primers used to produce PCR Amplicons for dsRNA Synthesis in An. gambiae. (0.03 MB DOC) [file pone.0011168.s005.doc]

| **Gene** | **Primer Sequence** |
| --- | --- |
| OXR1 | Fw 5'-AAAGCGACCCTGTTTGCGTC-3'  Rv 5'-TGACGAGCGTTTTGATGACGAAATC-3' |
| JNK | Fw 5'-TGCCAGGTCATACAGATGGA-3'  Rv 5'-GCCTGACTAGCCTTCAGTCG-3' |
| LacZ | Fw 5'-GAGTCAGTGAGCGAGGAAGC-3'  Rv 5'-TATCCGCTCACAATTCCACA-3' |
